# Death of Prof. Revere

**Published:** 1847-06

**Authors:** 


### DEATH OF PROFESSOR REVERE.

The eastern prints announce the death of Dr. John Revere, Professor of the Theory and Practice of Medicine in the medical department of the New York University. Dr. Revere began the study of medicine in Boston, of which he was a native, and after spending some years abroad, graduated in Edinburgh. He practised his profession for a time at Boston, and afterwards removed to Baltimore. During his residence in the latter city, he translated Magendie's Physiology, and published numerous papers on medical subjects. From Baltimore he went to Philadelphia, where he was appointed to the chair of Theory and Practice of Medicine in Jefferson Medical College. Subsequently he was chosen to fill the corresponding chair in the New York University. He died of pneumonia in New York city, on the 29th of April, aged 60 years.

Dr. Revere was a man of studious habits, of courteous and agreeable manners, and of an amiable disposition. His lectures were prepared carefully and with labor, and he had the reputation of being a successful teacher. His death is a loss to the profession, and especially to the school with which he was connected.

